# Community-acquired pneumonia-causing bacteria and antibiotic resistance rate among Vietnamese patients: A cross-sectional study

**DOI:** 10.1097/MD.0000000000030458

**Published:** 2022-09-09

**Authors:** Hung Do Tran, Yen Thi Bach Nguyen, Trung Thanh Tran, Trang Thi Thu Le, Ha Thi Thu Nguyen, Chau Minh Nguyen, Hop Thi Bach Le, Tham Thi Ngoc Phan, Tuyen Thi Thanh Vo, Hieu Thi Ngoc Bui, Vi Tuong Mai, Navy Yong, Thang Nguyen, Hung Gia Tran

**Affiliations:** a Can Tho University of Medicine and Pharmacy, Can Tho, Vietnam; b Vinh Long Province General Hospital, Vinh Long, Vietnam.

**Keywords:** antibiotic resistance, bacteria, community-acquired pneumonia, factor, pneumonia, Vietnam

## Abstract

Due to the overuse of antibiotics in treatment and regional variation in disease factors, community-acquired pneumonia (CAP) has a relatively high morbidity and mortality rate. This study determined the prevalence of bacteria that cause CAP and the rate of antibiotic resistance. From April 2018 to May 2019, a cross-sectional study was conducted on 254 CAP patients at hospitals and medical centers in the province of Vinh Long. Based on interviews and medical records, SPSS 18.0 was used to analyze the data. CAP-causing bacteria, antibiotic susceptibility, and extended-spectrum β-lactamase production of bacteria were determined by performing Identification and Antibiotic Susceptibility Testing on sputum specimens using the VITEK 2 Automated instrument. With a total of 254 patients, the age of 60s accounted for the highest prevalence. *Streptococcus pneumonia* was the leading factor, accounting for 12.6%, followed by *Klebsiella pneumonia* and *Pseudomonas aeruginosa* at 12.2% and 8.3%, respectively. The Enterobacteriaceae group was the highest at 36.5%, followed by other gram-negative bacteria (34%) and gram-positive bacteria (29.5%). Amoxicillin/clavulanic acid ranked the highest in antibiotic resistance, accounting for 31.4% of Enterobacteriaceae and 91.7% of non-Enterobacteriaceae. *S. pneumonia* resisted erythromycin at a high prevalence (84.4%), followed by clindamycin (71.9%) and tetracycline (78.1%). The age of 60s was the leading group in community pneumonia and had increased resistance to amoxicillin/clavulanic acid and cefuroxime.

## 1. Introduction

Community-acquired pneumonia (CAP) is an infectious disease with a high rate of morbidity and mortality globally, and it is especially prevalent among children and the elderly.^[1,2]^ To reduce mortality and aid physicians in diagnosing and prescribing the most effective antibiotics for patients, hospitals and medical centers have implemented a variety of innovative medical testing technologies and updated treatment guidelines. Continuous bacterial mutation complicates antibiotic resistance, making CAP research an alluring topic for scientists in Vietnam and around the world.

A systematic study about CAP in adults from 48 studies with 10,423 patients synthesized from multiple countries in Asia except the Middle East region. Significant infectious factors included *Streptococcus pneumonia, Mycoplasma pneumonia, Chlamydia pneumonia, Legionella spp.*, and *Haemophilus influenza*. However, compared with the studies from Western countries, *S. pneumonia* was less popular.^[3]^ Ronga et al (2019) made a retrospective analysis from 2015 to 2018 on *S. aureus* revealed that higher resistance rates were detected for penicillin, followed by oxacillin, levofloxacin, erythromycin, and clindamycin.^[4]^ In contrast, vancomycin, tigecycline, teicoplanin, linezolid, and daptomycin belonged to the lowest rate group.^[4]^ In Vietnam, a study by Le^[5]^ on 234 CAP patients, including 120 males and 114 females, recorded *S. pneumonia* as the main factor (50.6%), followed by *Pseudomonas aeruginosa* (16%) and *Acinetobacter baumannii* (14.8%). *S. pneumonia* was more resistant to β-lactam and less resistance to vancomycin.^[5]^ The resistance of *A. baumannii*, *K. pneumoniae*, and *P. aeruginosa* variated in other investigations.^[6]^

Depending on the region, time period, and topic of each study, bacterial species would vary. In addition, the overuse of antibiotics may cause complications in the treatment of CAP, such as decreased antibiotic efficacy, longer hospital stays, and even postrecovery complications. This study was conducted to determine the prevalence of CAP-causing bacteria and antibiotic resistance in patients examined at hospitals and medical centers in Vinh Long province, Vietnam, to assess antibiotic resistance in the region and develop an appropriate treatment regimen.

## 2. Materials and Methods

### 2.1. Study setting

We conducted a prospective and cross-sectional study involving 254 patients with CAP from 3 hospitals and 3 medical centers in Vinh Long province, Vietnam, from April 2018 to May 2019. Particularly, Vinh Long General Hospital was a second-class hospital with 600 beds, 29 departments, and a team of experts comprised of over 100 university-trained personnel, including medical doctors, pharmacists, and modern equipment. In 2016, the hospital treated 322,617 patients as inpatients and outpatients.

### 2.2. Patients and data collection

Patients over the age of 15 years who were diagnosed with CAP based on the guidelines of the American Thoracic Society and Infectious Diseases Society of America and treated for the first 36 hours of hospitalization met the eligibility requirements.^[7]^ To evaluate Severity-of-illness scores, the confusion, uremia, respiratory rate, low blood pressure, age 65 years or greater (CURB-65) criteria were applied.^[8]^ Due to the fact that samples were collected from multiple centers, with varying levels of healthcare, in CURB-65 criteria, we assessed confusion, respiratory rate, low blood pressure, and age ≥65 years. Before the onset of CAP symptoms, these individuals were not hospitalized and received no medical care for fourteen days. Patients consented to participate in the research. Exclusion criteria included patients with a history of human immunodeficiency virus (HIV) infection or HIV infection detected during the examination; patients with tuberculosis or receiving treatment for tuberculosis; and patients with pulmonary edema or pulmonary embolism.

According to a previous study conducted in Vietnam, the rate of bacteria isolation from sputum samples ranged from 40% to 70%.^[9]^ We anticipate this study to identify the bacterial cause for forty percent of the cases. We calculated the sample size for the study using the World Health Organization sample size calculation program, version 2.00, with α = 0.05 and ε = relative accuracy (0.25) = 1.96. The calculation yielded a sample size of 93 samples. However, the study was conducted in several hospitals, and in order to balance the research budget, we decided on the design coefficient k = 1.5. Consequently, the number of items determined by the preceding formula will be multiplied by 1.5. Therefore, minimum sample size of 140 patients should be utilized. In reality, 254 participants were enrolled in this investigation.

**Figure 1.**
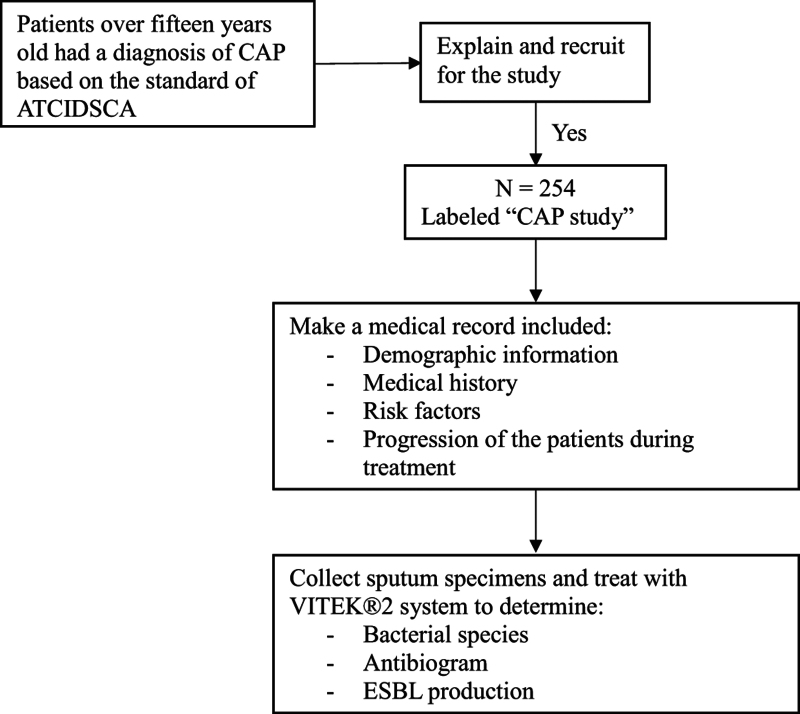
Schema of data collection. The sample size was determined using the World Health Organization (WHO) sample size computation version 2.00, with α = 0.05 and ε= relative accuracy (0.25) = 1.96. The calculation yielded a sample size of 93 samples. In fact, 254 samples were gathered for our investigation.

Data included demographic information, risk factors of pneumonia, clinical and subclinical characteristics based on interview, and medical records. We worked with VITEK^®^ 2 system to identify CAP-causing bacteria, antibiotic susceptibility, and extended-spectrum β-lactamase production of bacteria. All specimens of patients were collected and immediately sent to the Laboratory Department of Vinh Long General Hospital to assess the reliability of Bartlett standard (≥25 neutrophils and ≤ 10 squamous cells × 100). Confidence specimens were cultured using quantitative methods. We did not perform testing on atypical bacterial species. Sputum specimen was labeled “CAP study” in the request form and patient vial and was processed within 2 hours after collection (Fig. 1).

### 2.3. Statistical analysis

Data were analyzed using the SPSS program. The Chi-square test was used to compare the differences of ≥2 ratios between groups of patients with similar study characteristics. The compared mean values of ≥2 groups with a nonstandard distribution were evaluated by Kruskal-Wallis statistics. The comparison was significant when *P* < .05.

### 2.4. Ethical approval

This study has been approved by the Ethics board of General Hospital of Vinh Long Province and the Department of Science and Technology of Vinh Long Province. Patients or their legal representatives had been informed of the study’s purpose, method, risks, and benefits.

## 3. Results

### 3.1. Characteristics of the patients

A total of 254 patients participated, of which 85% were over 60 years old and 51.2% were female. The typical age was 73 years. The youngest was 16 years old, and the oldest was 98 years old. According to the CURB-65 scale, 70.5% of patients were over the age of 65 years, and the majority belonged to group 2 (62.6 %). Among individuals with a medical history, 33.1% had gastrointestinal ulcers, followed by diabetes (23.3%), minor liver disease (17.3%), and no occurrences of malignancy. Fever and fatigue are the most common systemic symptoms in patients with CAP, accounting for 77.2% and 71.7%, respectively (Table 1).

**Table 1:** General information of the participants.

Characteristics	Frequency (N = 254)	Proportion (%)
**Age group**		
Median	73 years old	
Lowest	16 years old	
Highest	98 years old	
< 60 years old	38	15.0
≥ 60 years old	216	85.0
**Gender**		
Male	124	48.8
Female	130	51.2
**Criteria to categorize the disease with CURB-65**		
≥ 65 years old	179	70.5
Confusion	3	1.2
Breathing rhythm ≥ 30 times/min or SpO_2_ < 90%/normal air	38	15.0
Systolic blood pressure ≤ 90 mm Hg	2	0.8
**Categorizing the disease to CURB-65**		
Group 1	64	25.2
Group 2	159	62.6
Group 3	31	12.2
**Medical history**		
Myocardial infarction	10	3.9
Congestive heart failure	9	3.5
Diabetes	59	23.3
Stroke	21	8.3
Hemiplegic	5	2.0
Dementia	22	8.7
COPD	20	7.9
Peptic ulcer disease	84	33.1
Gentle hepatic failure	44	17.3
Severe hepatic failure	1	0.4
Chronic kidney disease	21	8.3
Cancer	0	0.0
**Using corticosteroids before hospitalization**	3	1.2
**Using antibiotics within 3 mo before hospitalization**	17	6.7
**Using antibiotics after having symptoms before hospitalization**	19	7.5
**Smoking**	59	23.2
**Smoking at the moment**	10	3.9
**Drinking alcohol**	4	1.6
**Systemic symptoms**		
Confusion	4	1.6
Fatigue	182	71.7
Fever	196	77.2
**Symptoms of respiratory function**		
Apnea	113	44.5
Dyspnea	75	29.5
Opaque expectoration	252	99.2
Coughing	217	85.4
**Physical symptoms**		
Moist rales or dry rales	253	99.6
**Diagnostic signs**		
Severe pleural effusion on ultrasound	0	0.0
Other abnormal signs on ultrasound	135	53.1
Chest X-ray suited with CAP	252	99.2
**Laboratory finding**		
Neutrophils > 10 × 10^9^/L or < 4 × 10^9^/L	196	77.2

CAP = community-acquired pneumonia, COPD = Chronic Obstructive Pulmonary Disease, CURB-65 = confusion, uremia, respiratory rate, low blood pressure, age 65 years or greater,

### 3.2. Prevalence of CAP-causing bacteria

Positive cultures were found in 61.8% of the sputum samples. *Streptococcus pneumonia* was the leading factor, accounting for 12.6% of all participants, followed by *Klebsiella pneumonia* and *Pseudomonas aeruginosa* at 12.2% and 8.3%, respectively. The Enterobacteriaceae group accounted for 36.5% of the 156 positive specimens, followed by gram-negative bacteria (34%) and gram-positive bacteria (29.5%). Enterobacteriaceae had the highest proportion (54.4%), whereas *Enterobacter aerogenes* had the lowest (1.7%). Streptococcus pneumonia was the etiology of 69.6% of the gram-positive group. (Table 2).

**Table 2:** Sputum culturing results and prevalence of CAP-causing bacteria.

Characteristics	Frequency (n)	Proportion (%)
**Sputum culture**		
Negative	97	38.2
Positive	157	61.8
**CAP-causing factors**		
Enterobacteriaceae		
*Klebsiella pneumonia*	31	54.4
*Klebsiella pneumonia ESBL (+*)	5	8.8
*Escherichia coli*	4	7.0
*Escheriachia coli ESBL (+*)	7	12.3
*Enterobacter cloacae*	7	12.3
*Enterobacter aerogenes*	1	1.7
*Proteus mirabilis*	2	3.5
Non-Enterobacteriaceae		
*Pseudomonas aeruginosa*	21	65.6
*Pseudomonas spp*	2	6.3
*Acinetobacter baumannii*	9	28.1
Others gram-negative		
*Haemophilus influenza*	20	95.2
*Moraxella catarrhalis*	1	4.8
Gram-positive		
*Streptococcus pneumonia*	32	69.6
*Staphylococcus aureus*	2	4.3
*Staphylococcus aureus MRSA*	10	21.7
*Enterococcus faecalis*	2	4.3

CAP = community-acquired pneumonia, ESBL = extended-spectrum β-lactamase, MRSA = methicillin-resistant *Staphylococcus aureus*.

### 3.3. Antibiotic-resistant prevalence

Antibiotic resistance was highest for amoxicillin/clavulanic acid. There were 31.4% Enterobacteriaceae and 91.7% non-Enterobacteriaceae bacteria. Piperacillin/tazobactam demonstrated a high degree of sensitivity against gram-negative bacteria. Meropenem had a higher rate of antibiotic resistance in both the Non-Enterobacteriaceae and Enterobacteriaceae groups than imipenem, 6.3% versus 1.8% and 9.4% versus 3.5%, respectively (Table 3). *Streptococcus pneumonia* exhibited a high prevalence of erythromycin resistance (84.4%), followed by clindamycin (71.9%), and tetracycline (78.1%). Methicillin-resistant *Staphylococcus aureus* (MRSA) was resistant to all antibiotics, including erythromycin and clindamycin, but susceptible to doxycycline, tigecycline, vancomycin, and linezolid. (Table 4).

**Table 3:** Antibiotic resistance rate of gram-negative group.

		Gram-negative bacteria group		
		Enterobacteriaceae	Non-Enterobacteriaceae	Other
Beta lactam	Amox/Clavu	11 (31.4%)	22 (91.7%)	7 (36.8%)
	Ampi/Sul	3 (16.7%)	3 (50%)	3 (15%)
	Piper/Tazo	6 (10.5%)	4 (13.3%)	0
Cephalosporin	Cefuroxime	14 (40%)	8 (100%)	0
	Cefotaxime	19 (33.3%)	19 (61.3%)	0
	Ceftriaxone	19 (34.5%)	4 (100%)	0
	Ceftazidime	18 (31.6%)	3 (9.4%)	1 (5%)
	Cefepime	17 (30.9%)	38 (69.1%)	0
Carbapenem	Imipenem	2 (3.5%)	3 (9.4%)	0
	Meropenem	1 (1.8%)	2 (6.3%)	0
Aminoglycoside	Gentamycin	9 (15.8%)	2 (6.2%)	0
	Amikacin	0	2 (9.1%)	0
Quinolon	Ciprofloxacin	15 (26.3%)	6 (18.8%)	2 (9.5%)
	Moxifloxacin	3 (12%)	1 (9.1%)	0
Cyclin	Doxycycline	0	6 (58.7%)	2 (9.5%)

**Table 4:** Prevalence of antibiotic resistance.

	*Klebsiella pneumonia*	*Klebsiella pneumonia*	*Escherichia coli*	*Escherichia coli*	*Enterobacter cloacae*	*Pseudomonas aeruginosa*	*Acinetobacter baumannii*	*SAU MRSA*	*Streptococcus pneumoniae*	*HI*
	*ESBL (−*)	*ESBL (+*)	*ESBL (−*)	*ESBL (+*)						
Ampicillin	100	100	100	100	100	100	0			84.2
Penicillin									65.4	
Amox/Clavu	6.7	75	25	20	100	100	100			38.9
Piperacillin	94.1	100	0	100	0	0	0			0
Piper/Tazo	3.2	20	0	42.9	14.3	19	0			0
Ampi/Sul	7.1	100		100	100	100	0			15
Cefoxitin	4.8	100	25	100	100	100				
Cefuroxime	5.6	100	50	100	100	100	100			35
Cefotaxime	6.5	100	75	100	28.6	100	0		37.5	0
Ceftriaxone	6.7	100	75	100	33.3	100	0		34.4	0
Ceftazidime	6.5	100	75	100	14.3	13	0		0	5
Cefepime	6.7	100	75	100	0	13.6	0			
Ciprofloxacin	3.2	80	75	85.7	14.3	26.1	0	90		10
Levofloxacin	0				0	0	0	90	53.1	0
Moxifloxacin									43.5	
Gentamycin	0	60	25	57.1	14.3	8.7	0	80		
Amikacin	0	0	0	0	0	9.5	0			
Imipenem	0	20	0	0	0	13	0		0	0
Meropenem	0	20	0	0	0	8.7	0			0
Trime/Sulfa	41.9	100	75	57.1	57.1	100	22.2	70	34.4	60
Tetracycline	0					100		44.4	78.1	
Doxycycline	0		0			100	0	0		5
Tigecycline	0	20	0	0	0	100	0	0	0	
Oxacillin								100		
Erythromycin								100	84.4	
Clindamycin								100	71.9	
Vancomycin								0	0	
Linezolid								0	0	
Azithromycin										0
Colistin	0	0	0	0	0	0	0			
Rifampin								0		6.3

ESBL = extended-spectrum β-lactamase, MRSA = methicillin-resistant *Staphylococcus aureus*.

## 4. Discussion

The research was conducted on 254 CAP patients, 156 of whom had a positive sputum culture. Overall, *Streptococcus pneumonia* accounted for 12.6% of cases, followed by *Klebsiella pneumonia* (12.2%) and *Pseudomonas aeruginosa* (8.3%) The Enterobacteriaceae group accounted for 36.5% of the 156 positive specimens, followed by gram-negative bacteria (34%) and gram-positive bacteria (29.5%). In the Enterobacteriaceae family, *Klebsiella pneumonia* accounted for the largest proportion (54.4%), while *Enterobacter aerogenes* accounted for the smallest proportion (1.7%). 69.6% of the gram-positive group was caused by *Streptococcus pneumonia*. Amoxicillin/clavulanic acid was one of the most widely used antibiotics and had the highest prevalence of resistance in the β-lactam group, 31.4% in the Enterobacteriaceae group, and 91.7% in the non-Enterobacteriaceae group. Almost every antibiotic exhibited a higher rate of resistance to non-Enterobacteriaceae bacteria than to Enterobacteriaceae bacteria. *Streptococcus pneumonia* exhibited a high prevalence of erythromycin resistance (84.4%), followed by clindamycin (71.9%), and then tetracycline (78.1%). MRSA was resistant to all antibiotics, including erythromycin and clindamycin but sensitive to doxycycline, tigecycline, vancomycin, and linezolid.

There were 48.8% of males and 51.2% of females, indicating a relatively equal distribution. This was a remarkable aspect of this study, whereas others varied considerably.^[5,9]^ The typical age was 73 years. The youngest was 16 years old and the oldest was 98 years old. 85% of the participants were concentrated in the group of individuals older than 60 years of age. Due to comorbidities, nutritional disorders, and age-related swallowing disorders, the number of elderly patients with CAP has been on the rise, according to recent research.^[10]^ 33.1% of patients with a medical history had gastrointestinal ulcers, followed by diabetes (23.3%), mild liver disease (17.3%), and no cancer cases. Fever and fatigue are the most common systemic symptoms in patients with CAP, accounting for 77.2% and 71.7%, respectively. In the study by Musher and Thorner,^[11]^ ≈80% of CAP patients had fever, which was less common in elderly patients, and the body temperature typically dropped in the morning due to changes in body temperature throughout the day. In this study, peptic ulceration was the most prevalent (33.1%). Microbiological studies have demonstrated that a gastric pH > 4 is necessary for the overgrowth of gram-negative bacteria but not gram-positive bacteria.^[12]^ In addition to fever and fatigue, the prevalence of 2 other common CAP symptoms was also high: 77.2% and 71.7%, respectively.

According to results of the VITEK 2 system, the positive sputum culture prevalence was 61.4%, of which the 3 highest factors were *Streptococcus pneumoniae* (12.6%), *Klebsiella pneumoniae* (12.2%), and *P. aeruginosa* (8.3%). CAP-causing factors varied in comparison with other countries. Typically, the proportion of *S. pneumoniae* in Asia was lower than in Europe, 13.3% versus 25.9%.^[3]^ *S. pneumoniae* was the leading pathogenic bacteria through the systematic review and meta-analysis of Ghia et al^[13]^ on 1435 participants with *S. pneumoniae* infection was 19% (95% CI = 12%–26%; I^2^ = 94.5%, *P* < .01). Additionally, this ratio ranged from 18.3% to 19.05% in other studies.^[14,15]^ A study in Egypt from September 2015 to March 2017 noted that *K. pneumonia* was the main cause of CAP in this country, followed by *S. pneumonia* and *P. aeruginosa* with 7.78% for each, atypical bacteria appeared in 36 cases.^[16]^ The prevalence of the Khalil MM study was higher than that of Rehab H El-Sokkary, which could be explained by the effectiveness of the vaccine against *S. pneumonia* in Egypt or the low sensitivity of their identifying method.^[17,18]^ Among Indian CAP, adolescents and adult pathogenic bacteria included *K. pneumoniae* (1.6%-24.0%), *S. aureus* (1.0%–12.8%), *P. aeruginosa* (0.83%–11.6%), *Escherichia coli* (0.83%–8.57%), *Acinetobacter* spp. (0.83%–5.0%), and *Enterobacter* spp. (0.83%–4.0%).^[13]^ In Vietnam, *Streptococcus spp.* was one of the most popular CAP-causing bacteria in 11 countries of the Asian Network for Surveillance of Resistant Pathogens.^[19]^ A study in Ho Chi Minh city (2016–2017) identified the pathogenic bacteria by quantitative sputum culture and real-time polymerase chain reaction (PCR) detected *S. pneumoniae* (16.4%), *H. influenza* (9.6%), and *M. catarrhalis* (1.4%).^[20]^ Moreover, the research of Le^[5]^ on 234 CAP patients gave a remarkably high rate of *S. pneumonia* (50.6%), other species resembled our results, such as *P. aeruginosa* (16%), *A baumannii* (14.8%), Enterobacteriaceae (11.1%), and *S. aureus* (7.4%). Nowadays, *S. pneumoniae* has still been a common CAP-causing factor in Vietnam and other countries.

The traditional microbiological method is essential for identifying infectious disease-causing pathogens. Nonetheless, this technique may be less sensitive, resulting in inaccurate results, particularly with sputum samples. Due to the fact that sputum samples are inherently contaminated by the oropharynx, culturing the correct pathogenic bacteria is a significant challenge. Additionally, the most prevalent pathogenic bacteria that cause lower-respiratory disease are difficult to cultivate. In addition to sputum analysis, blood samples were also used to determine the causes of CAP. Although *S. pneumoniae* isolation from sputum may represent colonization and overestimate its role in CAP, the rate of *S. pneumoniae* as a cause of CAP was underestimated due to the insensitivity of the isolation technique from blood.^[13]^ Recent studies have demonstrated that real-time PCR is the most effective method for identifying pathogenic agents in pneumonia and lower-respiratory infectious diseases.^[19–23]^ In this study, the VITEK 2 system provided accurate and sensitive results. In addition, there was no difference in positive prevalence when compared with the conventional culture technique. This system could be technically and economically feasible in reality. Traditional microbiological methods based on biochemical reactions in each type of test tube posed a high risk of infection, whereas sample processing with the VITEK 2 system was completely automated. The results of the VITEK 2 system were typically available within 8 hours, allowing the physician to prescribe or alter the appropriate antibiotics promptly.

Amoxicillin/clavulanic acid was one of the most popular antibiotics and had the highest prevalence of resistance in the β-lactam group, of which 31.4% in Enterobacteriaceae group and 91.7% in non-Enterobacteriaceae group. In A cross-sectional study by Teklu et al^[24]^ on 426 samples of Enterobacteria, the highest level of resistance was reported to sulfamethoxazole/trimethoprim (77.0%), followed by amoxicillin/clavulanic acid (71.6%), cefotaxime (62.2%), cefepime (60.3%), ceftazidime (60.8%), and norfloxacin (58.8%). These results indicated that the rate of resistance to commercially available as well as commonly used drugs was becoming alarming in Ethiopia.^[24]^ Furthermore, it was possible to explain that the antibiotic therapy and antibiotic susceptibility of bacteria differ in different countries. piperacillin/tazobactam still well responded in the Enterobacteriaceae (89.5%) and Non-Enterobacteriaceae (86.7%). Our outcomes were lower than the study of Uc-Cachón et al^[25]^ in Mexico with high resistance to ampicillin (95.58%), cefuroxime (84.17%), and piperacillin (82.93%). This study revealed that non-Enterobacteriaceae group had a higher resistance rate than Enterobacteriaceae group in the carbapenem group. Similar to the Centers for Diseases Control and Prevention report, the rate of resistance to Enterobacteriaceae increased from 1% to 4% (2001–2011) and *K. pneumonia* resisted carbapenem developed from 2% to 10%.^[26]^ Besides, we found that *S. aureus* was significantly resistant to many antibiotics, including levofloxacin (75%), ciprofloxacin (75%), and moxifloxacin (66.7%) of the fluoroquinolone group. The resistance rates of *S. aureus* to erythromycin and azithromycin were up to 91.7% and 90%, respectively. Meanwhile, *S. pneumonia* resisted erythromycin with 84.8%. Luan et al’s^[27]^ research revealed a significant resistant proportion for *S. aureus*, but our study’s resistant rate was much higher than in Luan et al’s^[27^ (9.1% versus 84.8%). A study was conducted in the period 2004 to 2013 and showed that an increase in antibiotic resistance of *S. pneumonia*, such as levofloxacin from 0% to 16.7%, ciprofloxacin from 28% to 41%, erythromycin from 45.5% to 73.4%.^[28]^ Therefore, multidrug-resistant organisms have appeared in the hospital environment as well as in the community.

Our study was merely cross-sectional, with a primary emphasis on describing characteristics and prevalence. We did not identify the associated factors or the invention strategy. In addition, the number of cases did not adequately represent the community. Also, with a large number of samples collected from 3 hospitals and 3 medical centers, biases in diagnosis and treatment were inevitable due to the varying quality of healthcare. Therefore, more studies should be conducted in the future to develop more specific strategies for clarifying and resolving the restricted issues.

## 5. Conclusions

This study revealed that *Streptococcus pneumoniae* is the primary agent responsible for CAP. Amoxicillin/clavulanic had the highest rate of resistance among β-lactams. Non-Enterobacteriaceae exhibited greater resistance to carbapenem than Enterobacteriaceae. *Staphylococcus aureus* was extremely resistant to erythromycin (91.7%) and azithromycin (90%). Our research could assist clinicians in this region in selecting an appropriate antibiotic for a given situation. Despite the advancement of medical science, certain bacterial species have developed the ability to resist antibiotics. Therefore, community-acquired pneumonia remains a concern in the province of Vinh Long, Vietnam, and globally. In addition, we anticipated future research that could identify the relevant factors and provide clinical interventions to alleviate the problem of antibiotic resistance.

## Author contributions

Conceptualization, H.D.T. and Y.T.B.N.; methodology, formal analysis, investigation, H.D.T. and Y.T.B.N.; writing original draft preparation, M.T.V., Y.N., and H.G.T.; writing: review and editing, H.G.T, M.T.V., and T.N.; visualization, M.T.V.; supervision, H.D.T. All authors (H.D.T., Y.T.B.N., T.T.T., T.T.T.L., H.T.T.N., C.M.N., H.T.B.L., T.T.N.P., T.T.T.V., H.T.N.B., M.T.V., N.Y., T.N., and H.G.T.) have read and agreed to the published version of the manuscript.
